# Genomic analysis of multidrug-resistant *Delftia tsuruhatensis* isolated from raw bovine milk

**DOI:** 10.3389/fmicb.2023.1321122

**Published:** 2024-01-04

**Authors:** Pavel A. Andriyanov, Daria D. Kashina, Alena N. Menshikova

**Affiliations:** Federal Research Center for Virology and Microbiology, Branch in Nizhny Novgorod, Nizhny Novgorod, Russia

**Keywords:** *Delftia tsuruhatensis*, antimicrobial resistance (AMR), genomics, raw milk, phylogenomic analysis

## Abstract

*Delftia tsuruhatensis* is a gram-negative, aerobic bacterium mostly known as an organic pollutant degrading and growth-promoting microorganism. However, it recently emerged as an opportunistic human pathogen. To date, the source of *D. tsuruhatensis* infection is not clear. The majority of studies of *D. tsuruhatensis* have focused on environmental or clinical strains, while investigations of *D. tsuruhatensis* strains isolated from food sources are limited. In the present study, we report the case of *D. tsuruhatensis* isolation from raw bovine milk. Classical bacteriology approaches, as well as next-generation sequencing and comparative genomics, were used to characterize the features of the *D. tsuruhatensis* MR-6/3H strain. The MR-6/3H strain was resistant to 19 antimicrobials among 23 tested, including all aminoglycosides, phenicol, trimethoprim-sulfamethoxazole, and almost all β-lactams. Phylogenetically, the MR-6/3H was close to clinical origin strains, including those previously isolated in Russia. Comparative genomics revealed the presence of putative antimicrobial resistance genes in the MR-6/3H isolate, mostly associated with efflux systems. Notably, genus-specific OXA-926-like β-lactamase was also detected. In all, 27 putative virulence factors were predicted, the majority of which were associated with motility, adherence, stress survival, siderophore synthesis, and immunomodulation. In the MR-6/3H genome, the five prophage regions were identified, including two with intact levels. Integrons and CRISPR-Cas systems were not detected in the MR-6/3H isolate. Thus, our findings suggest that raw milk can be the potential source of and transmission route for the dissemination of multidrug-resistant *D. tsuruhatensis*.

## Introduction

*Delftia tsuruhatensis* is a gram-negative, motile, non-fermentative, aerobic bacterium. This species belongs to the *Comamonadaceae* family, order *Burkholderiales*, class *Betaproteobacteria*. *D. tsuruhatensis* was initially isolated from activated sludge collected from a domestic wastewater treatment plant in 2003 in Japan and described as a terephthalate-assimilating bacterium (Shigematsu et al., 2003). These bacteria are ubiquitous, but most frequently were isolated from polluted environments, rhizosphere soil, and activated sludge. Notably, plant growth-promoting effects of *D. tsuruhatensis* were observed. Strain HR4, isolated from the rhizoplane of rice (*Oryza sativa*) in China, was shown to suppress the growth of various plant pathogens (Han et al., 2005). Similarly, strain MTQ, isolated from the soil of the tobacco rhizosphere in China, also had antimicrobial activity against some phytopathogens (Hou et al., 2015). Interestingly, the antimicrobial and inhibitory activity of *D. tsuruhatensis* against such notable human ESKAPE pathogens was also reported (Malešević et al., 2019; Tejman-Yarden et al., 2019). Another remarkable feature of *D. tsuruhatensis* is the ability to biodegrade some organic pollutants, such as phenolic compounds, cyclopropylcarboxamides, terephthalate, etc. (Shigematsu et al., 2003; Liang et al., 2005; Zheng et al., 2007; Juárez-Jiménez et al., 2010; Juarez Jimenez et al., 2012; Ye et al., 2019). Thus, *D. tsuruhatensis* is interesting in terms of bioremediation, biotechnology, and new antimicrobial drug development.

*Delftia tsuruhatensis* was isolated from such animals as *Danio rerio* (Zebrafish: skin mucus) (Chaires et al., 2022) and *Oreochromis niloticus* (Nile tilapia: internal organs and injured eye) (Soto-Rodriguez et al., 2013; Preena et al., 2020), laboratory mice (inbred strain C57BL/6: proximal colonic tissue) (Saffarian et al., 2016), and even humans. *D. tsuruhatensis* was reported as an emergent opportunistic human pathogen. The first infection case was reported in 2011 by Preiswerk et al., where the catheter-related infection was observed in a 53-year-old woman from Switzerland. Moreover, isolates from collected blood had multidrug-resistant phenotype (MDR) and were resistant to ampicillin, some cephalosporins, all tested aminoglycosides, and colistin, whereas they were susceptible to amoxicillin-clavulanate, piperacillin-tazobactam, III generation cephalosporins, carbapenems, and fluoroquinolones. Ciprofloxacin-based therapy led to a positive infection outcome (Preiswerk et al., 2011). Afterward, some similar cases of *D. tsuruhatensis* infection were further reported, including infection in a premature infant (Tabak et al., 2013; Ranc et al., 2018; Cheng et al., 2021; Cho et al., 2021). Recent pan genomic investigations revealed some putative virulence factors in *D. tsuruhatensis* genomes, most of which were associated with nosocomial infections, for example, bloodstream infection, urinary tract infection, skin infection, etc. The multitude of antimicrobial resistance (AMR) genes were also predicted in some *D. tsuruhatensis* strains, including factors conferring resistance to aminoglycosides, fluoroquinolones, aminocyclitols, tetracyclines, phenols, and folate pathway antagonists (Yin et al., 2022). Furthermore, cases of AMR genes acquired via horizontal gene transfer (HGT) have been reported in *D. tsuruhatensis*. For instance, in the chromosome of the CRS1243 clinical strain, the *bla*IMP-1 beta-lactamase gene linked with mobile element conferring resistance to cephamycin, cephalosporin, penem, penam, and carbapenem was detected (Cho et al., 2021). Likewise, the TR1180 clinical strain was reported to harbor the unique 38-kb genomic island with the insertion of an In4-like integron containing some AMR genes (Cheng et al., 2021).

An open pan-genome with a great number of various proteins was also highlighted, which defines *D. tsuruhatensis* as a generalist pathogen with extensive genetic diversity and the ability to thrive in many ecological niches. Phylogenomic analysis revealed that *D. tsuruhatensis* strains appear to be particularly capable of animal colonizing and causing opportunistic infection (Bhat et al., 2022). Thus, it is important to investigate the possible sources of *D. tsuruhatensis*, as well as explore its genomic diversity and antimicrobial resistance mechanisms. In the present study, the case of *D. tsuruhatensis* isolation from unpasteurized milk (food source) is reported. We utilized common bacteriological methods, antimicrobial susceptibility evaluation, next-generation sequencing, phylogenomics, and comparative genomics approaches to comprehensively characterize the *D. tsuruhatensis* MR-6/3H strain.

## Results

### Bacterium isolation, cultural characteristics, and identification

**Figure 1:**
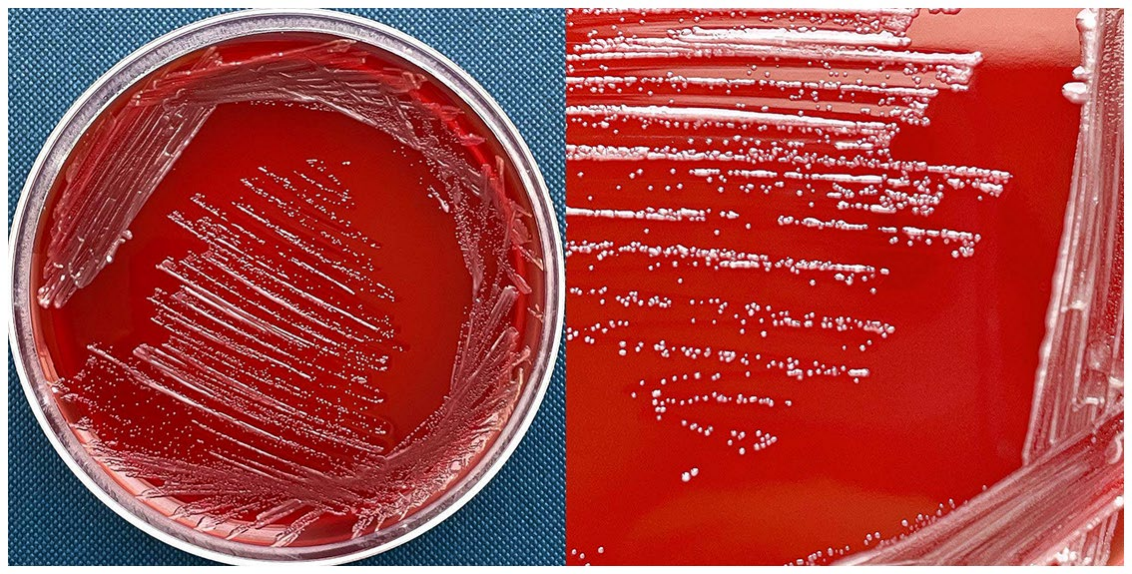
*D. tsuruhatensis* MR-6/3H strain on 5% human O-blood agar. MR-6/3H exhibited punctiform, circular, whitish colonies with a diameter of 0.5–2 mm andan entire margin and convex elevation; MR-6/3H had a gamma-hemolysis activity (absence of visible hemolysis).

*Delftia tsuruhatensis* MR-6/3H was isolated in February 2022 from raw bovine milk, from a retail seller in the territory of the Nizhny Novgorod region, Russia. On 5% human O-blood agar, MR-6/3H formed punctiform, circular, whitish colonies with a diameter of 0.5–2 mm and an entire margin and convex elevation (Figure 1). MR-6/3H exhibited Gamma-hemolysis on human O-group erythrocytes. On the tryptone soya agar (TSA), MR-6/3H exhibited the same colony morphology as on blood agar but had opaque colonies. The strain was positive for oxidase and catalase. Microscopic analysis revealed a typical Gram-negative, rod-shaped bacterium.

We used sequencing of the 16S rRNA gene for the isolate identification. The assembled consensus of the 16S rRNA gene was 1,389 bp in length with an identity percentage of 100% with sequences of both species: *D. tsuruhatensis* NBRC 16741 (T) (GenBank accession no. BCTO01000107) and *D. lacustris* LMG 24775 (T) (GenBank accession no. jgi.1102360). MR-6/3H also had 99.5% of sequence identity with *D. acidovorans* 2,167 (T). Further genomic approaches were used to clarify the species identification.

### Antimicrobial susceptibility profile of *Delftia* MR-6/3H isolate

Antimicrobial susceptibility testing was performed via the disk-diffusion method on 23 antimicrobials based on the european committee on antimicrobial susceptibility testing guidance (EUCAST v 12.02022). *Delftia* MR-6/3H was resistant to 19 antimicrobial drugs widely used in clinical practice including all aminoglycosides tested (gentamicin, amikacin, tobramycin, kanamycin, and neomycin), chloramphenicol, trimethoprim-sulfamethoxazole, and almost all β-lactams (ampicillin, amoxicillin-clavulanic acid, aztreonam, meropenem, imipenem, ceftobiprole, cefotaxime, cefepime, piperacillin, piperacillin-tazobactam, ticarcillin, and ticarcillin-clavulanic acid). The *Delftia* MR-6/3H strain was susceptible to ceftazidime, tetracycline, and both fluoroquinolones: ciprofloxacin, and levofloxacin. Such phenotype was considered multidrug-resistant (MDR). The susceptibility profile with zones of growth inhibition diameters and antibiotics tested are shown in Table 1.

**Table 1:** List of antibiotics tested, inhibition zone diameter, and susceptibility profile.

Antimicrobial class	Antibiotic	Inhibition zone diameter, mm	Interpretation
Aminoglycosides	Amikacin	0	R
	Gentamicin	0	R
	Kanamycin	11	R
	Neomycin	0	R
	Tobramycin	0	R
ß-lactams: Penicillins	Ampicillin	0	R
	Piperacillin	19	R
	Ticarcillin	0	R
	Amoxicillin-clavulanic acid	0	R
	Piperacillin-tazobactam	18	R
	Ticarcillin-clavulanic acid	17	R
ß-lactams: Cephalosporins	Cefepime	11	R
	Cefotaxime	15	R
	Ceftazidime	32	S
	Ceftobiprole	12	R
ß-lactams: Carbapenems	Meropenem	22	R
	Imipenem	29	R
ß-lactams: Monobactam	Aztreonam	16	R
Fluoroquinolones	Ciprofloxacin	33	S
	Levofloxacin	28	S
Other	Chloramphenicol	19	R
	Tetracycline	31	S
	Trimethoprim-Sulfamethoxazole	0	R

R - Resistant: A microorganism is categorized as “Resistant” when there is a high likelihood of therapeutic failure even when there is increased exposure; S - Susceptible, standard dosing regimen: A microorganism is categorized as “Susceptible, standard dosing regimen,” when there is a high likelihood of therapeutic success using a standard dosing regimen of the agent.

### Next-generation sequencing, assembly, and annotation

Assembly statistics are shown in Table 2. The assembly of the *Delftia* MR-6/3H genome contained 52 contigs with a total length of 6,468,367 bp (~6,4 Mb) and a GC content of 66.69%. The average read coverage was 315. In total 6,115 Open reading frames (ORFs) were detected by Rapid Annotation using Subsystem Technology (RAST), including 71 tRNA and 3 rRNA genes, 4,619 proteins with functional assignments, and 1,496 hypothetical proteins.

**Table 2:** General features and quality control of *Delftia* MR-6/3H genome assembly.

Attribute	Value
Number of contigs	52
Largest contig	883,805
Total length	6,468,367
GC (%)	66.7
N50	408,932
N75	182,482
L50	6
L75	11
ORFs	6,115
# N’s per 100 kbp	0
tRNA genes	71
rRNA genes	3
Proteins with functional assignments	4,619
Hypothetical proteins	1,496
CheckM2 Completeness (%)	100
CheckM2 Contamination (%)	0.13
CheckM2 Coding density	0.898
CheckM2 Average gene length (bp)	333.765

### Phylogenomic analysis

As the 16S rRNA gene sequencing method is unable to delineate between *D. tsuruhatensis* and *D. lacustris* species, gold standard approaches based on next-generation sequencing both Average Nucleotide Identity (ANI) and Type Strain Genome Server (TYGS) were used for further identification.

**Figure 2:**
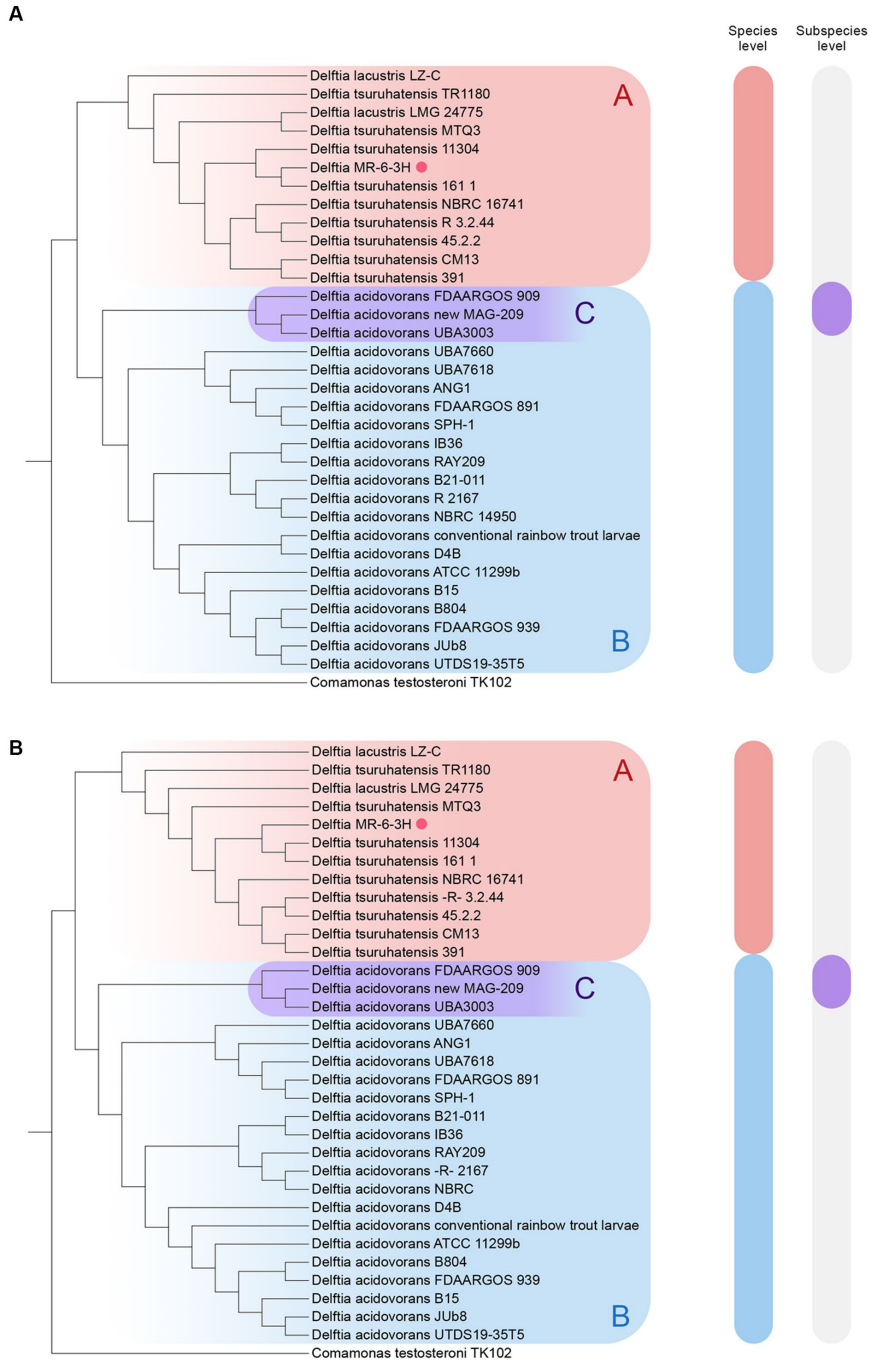
Reconstruction of the whole-genome cladograms of 33 Delftia spp. with *Delftia* MR-6/3H strain (labeled with pink circle): **(A)** TYGS cladogram. **(B)** ANI cladogram. A clade (Red-labeled) - *D. tsuruhatensis/lacustris*; B clade (blue-labeled) - *D. acidovorans*, C clade (violet-labeled) - putative *D. acidovorans* subspecies. The following type strains were used: *D. acidovorans* 2167 (T), *D. lacustris* LMG 24775 (T), and *D. tsuruhatensis* NBRC 16741 (T). *Comamonas testosteroni* TK102 was utilized as an outgroup.

**Figure 3:**
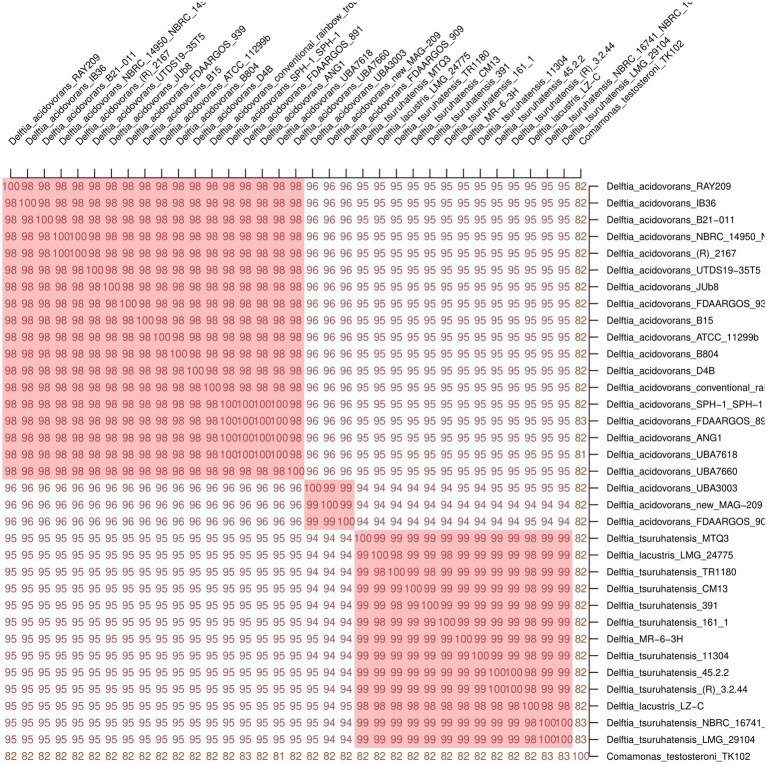
Average Nucleotide Identity (ANI) matrix of 33 *Delftia* spp. with *Delftia* MR-6/3H strain. As a cutoff value for species delineation >95% ANI was used. *Delftia* MR-6/3H strain formed a common cluster on the matrix with *D. tsuruhatensis-D. lacustris* species. Clusters with ANI ≥ 98 are marked in red: *D. acidovorans* cluster (on the top), putative *D. acidovorans* subspecies cluster (in the middle), and *D. tsuruhatensis-D. lacustris* cluster (at the bottom). *Comamonas testosteroni* TK102 was used as an outgroup.

The final cladograms are shown in Figure 2. The *Delftia* MR-6/3H strain clustered together with *D. tsuruhatensis* as a separate lineage (A clade) from *D. acidovorans* species clade (B clade). This topology was observed in both cladograms; it suggests that *Delftia* MR-6/3H (labeled with a pink circle) belongs to the *D. tsuruhatensis* species. Notably, being different species, *D. lacustris* and *D. tsuruhatensis* formed a single clade (A clade). Additionally, there was a putative subspecies clade inside the *D. acidovorans* cluster (C clade). These observations were supported by ANI matrix data (Figure 3) where ANI ranged between members of mentioned clades: 98–100% for the A clade and 99–100% for the C clade. The members of the latter clade had only 96% with other *D. acidovorans* strains and 99–100% between each other.

**Figure 4:**
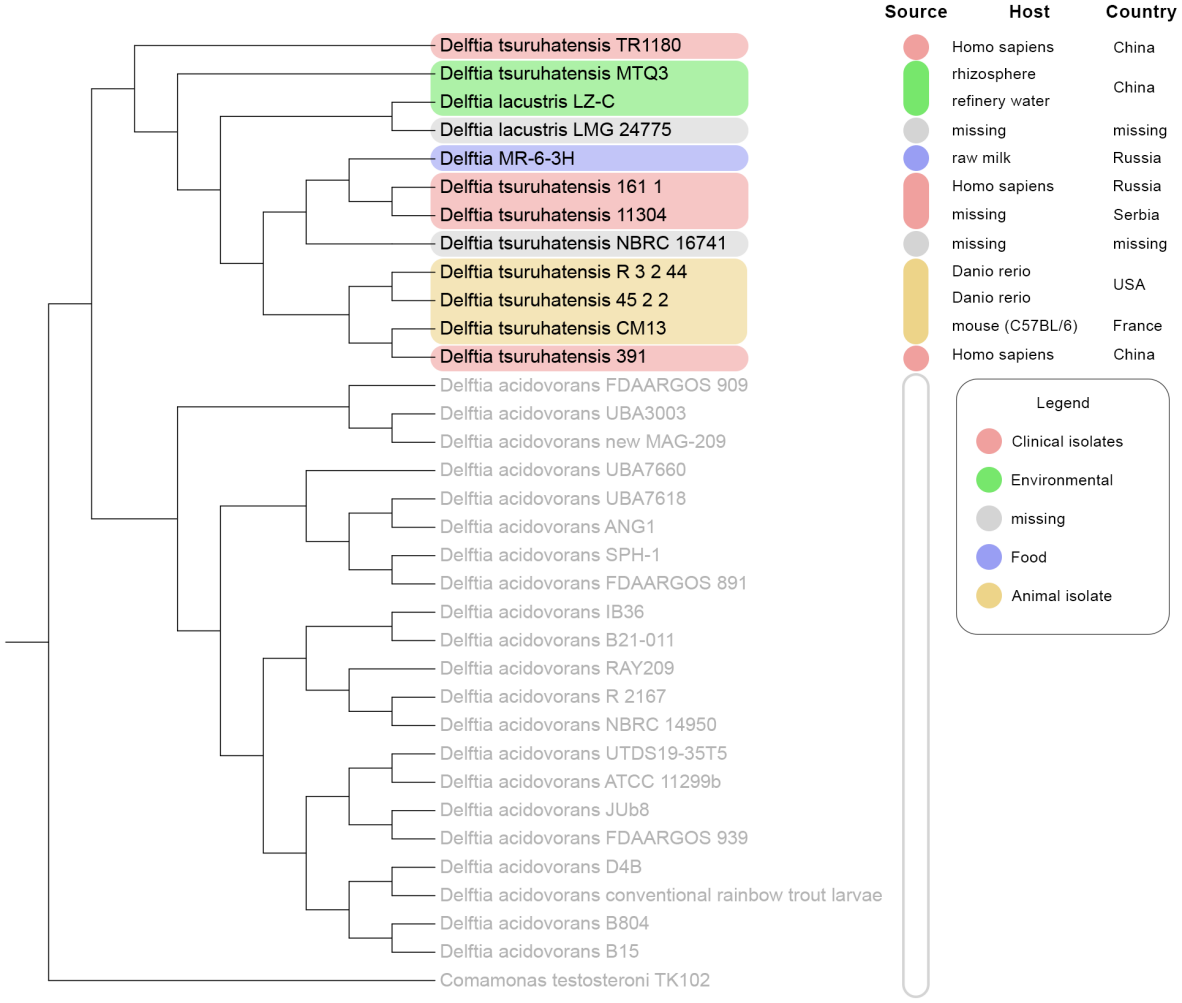
REALPHY-based phylogenomic analysis of 33 *Delftia* spp. strains: *D. tsuruhatensis*/*D. lacustris* clade is highlighted in colors depending on the source type. *D. acidovorans* 2167 (T), *D. lacustris* LMG 24775 (T), and *D. tsuruhatensis* NBRC 16741 (T) were used as reference genomes.

The members of the A clade (*D. tsuruhatensis/lacustris*), to which *Delftia* MR-6/3H belonged, were chosen for further investigation. To clarify the sophisticated phylogenetic relationships among *Delftia* spp. strains and *Delftia* MR-6/3H strain, the REALPHY web tool was utilized (Figure 4). In general, the REALPHY cladogram was almost identical to the ANI and TYGS ones. *Delftia* MR-6/3H formed a separate clade with *D. tsuruhatensis* 161/1 and *D. tsuruhatensis* 11304 strains. Interestingly, both of them have a clinical origin. Strain *D. tsuruhatensis* 161/1 was isolated from a human host in Samara, Russia in February 2020. Strain *D. tsuruhatensis* 11304 has a “missing” particular source (but has a clinical origin) and was isolated in Serbia in March 2019. The mentioned clade formed a common phylogeny cluster with *D. tsuruhatensis* NBRC 16741 (T) type strain. Strains with animal origin (3.2.44, 45.2.2, and CM13) clustered together with *D. tsuruhatensis* 391, which was isolated from a human host. Both of the abovementioned clades (*Delftia* MR-6/3H with *D. tsuruhatensis* 161/1 and *D. tsuruhatensis* 11304, *D. tsuruhatensis* NBRC 16741 (T) and strains with animal origin and *D. tsuruhatensis* 391) formed a single sister group.

### Comparative resistome analysis

Next-generation sequencing data were used to find putative antimicrobial resistance factors in 11 strains of *Delftia* spp., including the *Delftia* MR-6/3H strain (Supplementary Table S1). Antimicrobial resistance (AMR) gene homologs prediction was performed by BLASTp searching against the CARD database.

Among all 11 strains, 31 AMR gene homologs were predicted. Individually, each strain possessed from 11 to 17 AMR genes. All detected homologs were grouped into five subgroups regarding their resistance mechanism: efflux pumps, β-lactamases, aminoglycoside inactivation enzymes, diaminopyrimidine resistance, and others. The following factors were observed in all genomes tested, including *Delftia* MR-6/3H: efflux pumps (*ceoB*, *mexD*, *paer_soxR*, *adeB*, *mdtC*, *oprJ*, and *abeS*) and OXA-926-like β-lactamase. These genes confer resistance to a wide range of antimicrobials as well as disinfectants, for instance: penams, cephalosporins carbapenems, aminoglycosides, fluoroquinolones, phenols, diaminopyrimidine antibiotics, tetracycline, aminocoumarin, macrolide antibiotics, acriflavine antiseptic, and various disinfecting agents. In addition, the *Delftia* MR-6/3H strain harbored three additional efflux pump genes that were missing in some other strains: *oqxB*, *muxB*, and *mexC*. The latter is known to be involved in the extrusion of toxic substrates, including a wide range of antibacterials, but additionally, they can pump monobactams as well as nitrofuran compounds outside the cell.

Some *Delftia* spp. strains harbored unique AMR factors with high amino acid identity percentages that were not observed among other strains. For example, *D. tsuruhatensis* 391 possessed *sul1*, which confers resistance to sulfonamide antibiotics. *D. tsuruhatensis* TR1180 possessed also *sul1*, but additionally *dfrA16* (trimethoprim resistance), *aadA3* (aminoglycoside resistance), *cmlA9* (phenicol resistance), and *tet* (G) (tetracycline resistance). *D. lacustris* LZ-C harbored *cmx* (phenicol resistance), and *aph (3″)-Ib*, *aph (6)-Id* genes (aminoglycoside resistance).

### Comparative analysis of putative virulence genes

In total, 47 homologs of virulence factors (VF) were predicted among 11 *Delftia* spp. strains using BLASTp search against the Virulence Factor Database (VFDB) (Supplementary Table S2). Each strain possessed from 24 to 31 putative VF genes. All observed factors formed eight groups based on their virulence category: motility, adherence, immune modulation, stress survival, secretion system, siderophore, fitness, and others. Four motility-associated factors were detected in all 11 *Delftia* spp. strains (*cheY*, *fliP*, *fliI*, and *fliN*); these factors are responsible for flagellar biosynthesis, assembly, and function, which is consistent with the motility phenotype of *Delftia* species. The adherence group contained four genes common for all strains: *pilT*, *pilT2*, *pilG* (twitching motility proteins), and *htpB* (heat shock protein). Notably, three genes from the immune modulation category were observed among strains that had only clinical and animal (45.2.2 and CM13) origin, and were detected in the MR-6/3H isolate as well: *gmd* (GDP-mannose 4,6-dehydratase), *wzt* (O-antigen export system ATP-binding protein), and *per* (perosamine synthetase). Stress survival factors such as *katA* (catalase), *sodB* (superoxide dismutase), and *clpP* (ATP-dependent Clp protease) were also detected in all strains. Some genes related to the type IV secretion system were found among all genomes: *STM0274* (EvpB family type VI secretion protein), *vipA* (tubule-forming protein), and *clpV1* (type VI secretion system AAA+ family ATPase). Genes coding for siderophore transport were also predicted, among them two permease proteins (*bauD* and *bauC*), periplasmic siderophore-binding protein (*bauB*), and ferrienterobactin ABC transporter ATPase (*fepC*). Full sets of the abovementioned siderophore genes were found only in the following *Delftia* strains: MR-6-3H, 11304, 16741 (T), MTQ3, and LZ-C. Two fitness factors were identified only in the *D. tsuruhatensis* 391 strain, which was isolated from a human patient: *mtrD* (multiple transferable resistance system protein MtrD) and *acrB* (acriflavine resistance protein B). Additionally, two factors, namely, *adeG* (cation/multidrug efflux pump), which is related to biofilm formation, and *pvdF* (pyoverdine synthetase F), were observed among all *Delftia* spp. strains.

Compared to the strains with clinical origin (161/1, 11304, 391, and TR1180) the MR-6/3H strain was the closest to the 161/1 and TR1180 strains based on the VF gene set. Moreover, our strain harbored the *bauB* gene that was absent in the two abovementioned strains. The *bauB* gene codes for an additional periplasmic siderophore-binding protein (a part of a ferric siderophore ABC transporter). There were also three genes from the immune modulation category observed among strains that had only clinical and animal (45.2.2 and CM13) origin, but were present in the MR-6/3H isolate as well: *gmd* (GDP-mannose 4,6-dehydratase), *wzt* (O-antigen export system ATP-binding protein), and *per* (perosamine synthetase).

### Integron possessing analysis

Integrons are known as mobile genetic elements that contribute greatly to antimicrobial resistance dissemination. Integrons and their elements were predicted via IntegronFinder 2.0 among 11 *Delftia* spp. genomes (Supplementary Table S3). A complete integron sequence was detected in *D. tsuruhatensis* TR1180 only, which was isolated from a human patient. It consisted of an integrase gene (*int*), two I class promoters: Pint_1 (integrase promoter) and Pc_1 (cassette promoter), one *attI* and *attC* sites, and carried two gene cassettes (Figure S1a). CARD search showed that the first of them (NZ_CP045291.1_3624) is dihydrofolate reductase gene *dfrA16* and the second one (NZ_CP045291.1_3624) is aminoglycoside nucleotidyltransferase gene *aadA3*.

In the genome of *D. tsuruhatensis* 161/1, the CALIN (clusters of *attC* sites lacking integron-integrases) was detected. The latter was composed of three gene cassettes and two *attC* sites (Supplementary Figure S1). All of these proteins were annotated as unknown proteins (or hypothetical proteins) using BLASTp search against the GenBank protein database. We used HMMER search against reference proteomes as a more sensitive algorithm, especially in a homology search for unknown proteins, to identify their homology. Notably, HMMER search showed that one of the aforementioned proteins (NZ_JAKZJM010000052.1_2) matched with an unknown protein from the genome of the eukaryotic organism (fish) *Oreochromis niloticus* with an E-value of 5.5e-08. Gene Ontology (GO) annotation of this protein following the Unipro database showed that it is a putative cytoplasmic protein NLRC5 with an innate immune response function. The second protein (NZ_JAKZJM010000052.1_3) matched with an unknown protein from *Microvirga aerophila* with an E-value of 1.0e-16. The GO annotation for this protein was calcium-binding membrane protein with homophilic cell adhesion activity. The third one (NZ_JAKZJM010000052.1_4) matched with an unknown protein from *Sunxiuqinia dokdonensis* that had an e-value of 1.9e-37. The GO annotation for this protein was the ABC transporter protein.

In the chromosome of *D. lacustris* LZ-C, the CALIN was also found. It contained two *attC* and one gene cassette. This protein was matched with an unknown phage protein from *Pseudoalteromonas luteoviolacea* DSM 6061.

### Prophage and CRISPR-Cas search in the *Delftia* MR-6/3H genome

In the *Delftia* MR-6/3H genome, five prophage regions were identified. Among them, two had an intact level (scores of 150 and 100), one had a questionable level (score of 90), and two had an incomplete level (scores of 40 and 50). Two intact prophages had 46.5 Kb genome length with GC 64.82%, which had the closest match with *Burkholderia* phage BcepMigl, and 38 Kb genome length with GC 64.31%, which had the closest match with *Pseudomonas* phage JBD18. The BcepMigl-like prophage contained 32 CDS, among them integrase, portal proteins, fiber proteins, head proteins, and terminase as well as some phage-like proteins and hypothetical proteins that were annotated. The JBD18-like prophage contained 44 CDC, among them head proteins, tail proteins, phage-like proteins, and hypothetical proteins, and the absence of integrase was observed. Other prophages also contained different virion proteins and hypothetical proteins, but integrases were absent. CRISPR-Cas systems were absent in the MR-6/3H strain.

## Discussion

*Delftia tsuruhatensis* is known as a bacterium that can promote the growth of plants as well as biodegrade some organic pollutants in the environment, but also as an emergent opportunistic pathogen with natural resistance to some antimicrobials. In the present study, we report the case of multidrug-resistant *D. tsuruhatensis* isolation from unpasteurized bovine milk, collected in Russia. Classical microbiology methods, next-generation sequencing, and analysis approaches were applied to characterize our isolate.

Previously, strains of *D. tsuruhatensis* species were isolated from a wide range of sources such as active sludge (Shigematsu et al., 2003; Li et al., 2020), seawater (Juárez-Jiménez et al., 2010), soil (Zheng et al., 2007), rhizosphere (Guo et al., 2016), rice husk and swine manure (Chen et al., 2022), fish (*Danio rerio* and *Oreochromis niloticus*) (Preena et al., 2020; Chaires et al., 2022), mice (inbred strain C57BL/6) (Saffarian et al., 2016), and from human patients (Preiswerk et al., 2011; Tabak et al., 2013; Ranc et al., 2018; Cheng et al., 2021; Cho et al., 2021). Notably, the isolation of five *D. tsuruhatensis* strains from raw milk was also reported by Elionora Hantsis-Zacharov and Malka Halpern in 2007 in Israel (Accession no. EF204212 to-16) (Hantsis-Zacharov and Halpern, 2007). This indicates the ubiquitous nature of this bacterium. Recent phylogenomic investigations of the genus *Delftia* revealed that the members of the latter encode plenty of proteins (have open pan-genome) that allow them to succeed in many ecological niches (Bhat et al., 2022; Yin et al., 2022). However, information about microbiological and genomic analysis of *D. tsuruhatensis* strains isolated from food and raw milk is absent or limited. In our study, we cannot determine the certain source of the MR-6/3H isolate, because unpasteurized milk samples can be contaminated through various sources from the dairy cow itself to the hands of sellers. Yet, in the previous research work where five isolates of *D. tsuruhatensis* were isolated from raw milk, it was reported that milking was performed using modern automated milking facilities, and milk samples were taken under sterile conditions (Hantsis-Zacharov and Halpern, 2007). It reduces the internal contamination likelihood and may indicate that raw milk can be a source of *D. tsuruhatensis*. However, more investigations are needed to elucidate possible sources of this bacterium.

The 16S rRNA gene sequencing approach is recommended for the majority of bacterial species identification and delineation (Stackebrandt et al., 2002). However, we faced some ambiguities during identification via this method: strain MR-6/3H had the same identity percentage with both *D. tsuruhatensis* and *D. lacustris*. Similar observations were reported by investigators who dealt with *Delftia* identification, where 16S rRNA gene sequencing could not delineate between *D. tsuruhatensis* and *D. lacustris* (Preiswerk et al., 2011; Cheng et al., 2021). In our case, the abovementioned issue was resolved through ANI and TYGS, which are both gold-standard methods based on next-generation sequencing. Interestingly, in compliance with our analysis, *D. tsuruhatensis* and *D. lacustris* species formed a common clade. This observation is also supported by values of the ANI matrix as well as REALPHY phylogeny. The same findings were recently reported by Bhat et al. (2022). Our findings indicate that both *D. tsuruhatensis* and *D. lacustris* belong to the same species. This taxonomic confusion could arise because the aforementioned species were initially described based on 16S rRNA phylogeny, which cannot delineate species of the *Delftia* genus, and conventional DNA–DNA hybridization (DDH), which is error-prone and a significantly inaccurate method compared to *in silico* WGS approaches such as ANI and TYGS (Goris et al., 2007; Meier-Kolthoff and Göker, 2019). Similarly, we observed a subclade made up of three strains in the *D. acidovorans* species cluster in all three cladograms. Presumably, these strains can belong to the subspecies of *D. acidovorans*. The same observation was also reported recently (Bhat et al., 2022). We suggest that some species of the *Delftia* genus require taxonomic revision by the use of a polyphasic taxonomy approach. It is worth noting that the WGS approach is most effective when used in conjunction with other morphological, ecological, and molecular biology data.

*Delftia tsuruhatensis* is known as an emergent human opportunistic pathogen. To date, infections caused by this bacterium are known only in immunocompromised persons and newborns. *D. tsuruhatensis* can cause severe life-threatening infections, manifested primarily as pneumonia and catheter-related bloodstream infections (Preiswerk et al., 2011; Tabak et al., 2013; Ranc et al., 2018; Cheng et al., 2021; Cho et al., 2021). Interestingly, our *D. tsuruhatensis* MR-6/3H strain collected from raw milk was phylogenetically close to strains with clinical origin (*D. tsuruhatensis* 161/1 and *D. tsuruhatensis* 11304), and they formed a single sister group. Notably, *D. tsuruhatensis* 161/1 was isolated in Samara, Russia, in early 2020 from a human patient. *D. tsuruhatensis* 11304 strain was isolated in 2014 in Serbia, but a particular source was missing. Comparative analysis of putative virulence genes revealed a similar set of genes detected among the abovementioned strains. The genome of the MR-6/3H strain harbored genes that are related to flagella machinery function (*cheY*, *tufA*, *flgG*, *fliI*, and *fliN*), which well correlates with the motile phenotype of *Delftia* spp. Flagella is known as an important virulence factor, which serves for both motility and adhesion in such pathogens as *Pseudomonas aeruginosa* and *Escherichia coli* (Haiko and Westerlund-Wikström, 2013). Another group of genes (*tufA*, *pilT*, *htpB*, *pilT2*, and *pilG*) coding for adherence factors, including elongation factor Tu, twitching motility proteins (TMPs), and heat shock protein HtpB, were also predicted. For instance, TMPs were shown to be the major virulence factor of *P. aeruginosa*, which are involved not only in motility but also in invasion in a multilayer epithelial cell barrier (Alarcon et al., 2009). Lipopolysaccharide (LPS), also known as endotoxin, is a component of the outer membrane of gram-negative bacteria that contributes greatly to virulence due to its immunomodulatory and pro-inflammatory properties, including septic shock (Aurell and Wistrom, 1998). Three genes of LPS synthesis and excretion were detected in the *D. tsuruhatensis* MR-6/3H strain: *gmd*, *wzt*, and *per*. Other types of putative virulence genes were also observed in the MR-6/3H genome coding for type IV secretion system machinery, stress survival factors (catalase, superoxide dismutase, and urease), siderophores, etc. This indicates that MR-6/3H possesses a particular set of VFs that can confer virulence properties comparable to those of clinical origin strains: *D. tsuruhatensis* 161/1 and *D. tsuruhatensis* 11304. Notably, a similar set of putative virulence genes was detected in *D. tsuruhatensis* 11304 (Cheng et al., 2021). The genes mentioned above, coding for LPS synthesis and excretion, were observed only among strains with clinical or animal origin. Besides the immunomodulation effect that LPS has, the crucial role of this factor in animal and human colonization was also noted for such pathogens as *Aeromonas hydrophila* (Merino et al., 1996), *Salmonella Typhimurium* (Licht et al., 1996), *Helicobacter pylori*, and *P. aeruginosa* (Maldonado et al., 2016). Probably, an association of such LPS genes transports with animal and human origins of some strains is not accidental. Nonetheless, predicted virulence factors were detected using the protein homology model at the genotype level and need to be further experimentally investigated to show their contribution to the virulence of *D. tsuruhatensis*.

Antimicrobial resistance (AMR) is a crucial global health concern. A wide number of pathogens can acquire and exchange AMR genes, which leads to antimicrobial therapy failure and consequently to an increase in mortality (Murray et al., 2022). Antimicrobial resistance profile, particularly the multidrug-resistant (MDR) profile of clinical *D. tsuruhatensis* strains, was reported several times. In 2011, Preiswerk et al. reported the first case of *D. tsuruhatensis* infection, in which a blood-collected strain was resistant to ampicillin, I and II generation cephalosporins, all tested aminoglycosides, and colistin, but was susceptible to amoxicillin-clavulanate, piperacillin-tazobactam, III generation cephalosporins, fluoroquinolones, and carbapenems (Preiswerk et al., 2011). In another case reported in 2021, sputum specimen isolate TR1180 was resistant to β-lactams (ampicillin and cefazolin), all tested aminoglycosides, tetracycline, and trimethoprim-sulfamethoxazole based on MIC evaluation (Cheng et al., 2021). Both the abovementioned AMR profiles are quite similar. Our MR-6/3H strain had almost the same AMR phenotype, including resistance to almost all β-lactams, all aminoglycosides, chloramphenicol, and trimethoprim-sulfamethoxazole, but had susceptibility to ceftazidime, tetracycline, ciprofloxacin, and levofloxacin. In general, the AMR phenotype of the MR-6/3H strain correlated with the observed resistome, except for tetracycline and fluoroquinolones. Such discrepancy may be explained by impairments in some efflux systems encoded, misregulation of these genes, as well as their reduced expression. Alternatively, resistance to fluoroquinolones is usually related to point mutation in the *gyrA* gene (DNA gyrase) and with efflux to a lesser extent (Jacoby, 2005). Resistome analysis also revealed a resemblance between putative resistance factors, which were primarily made up of efflux genes, among different *Delftia* strains. Efflux pumps are capable of extruding a wide range of toxic compounds, including antibiotics. In 2021, Cong Cheng et al. reported similar resistance factors, including the variety of efflux pumps, when analyzing the multidrug-resistant *D. tsuruhatensis* TR1180 strain (*mexC*, *mexD*, *oprJ*, *muxB*, *oqxB*, *adeB*, *adeS*, *mdtC*, etc.) (Cheng et al., 2021). Apparently, the main intrinsic resistance mechanism of *Delftia* species is related to the regulation of membrane permeability and efflux systems, as is typical for *P. aeruginosa*, which uses multidrug efflux (Mex) systems and porins mainly (Pang et al., 2019). Additionally, we found the chromosome-encoded genus-specific β-lactamase, which was close to *Klebsiella* OXA-926 with an amino acid sequence identity of 70–73%. The latter was identified in 2021 from *Klebsiella pneumoniae* ST29 (Liu et al., 2021). OXA belongs to the class D β-lactamases, which have various substrate specificities against β-lactams but also can be mobilized by plasmids (Sawa et al., 2020). OXA-926 was reported to confer reduced susceptibility to piperacillin, piperacillin-tazobactam, and cephalothin as well as partial resistance to avibactam. The particular substrate specificity of *Delftia*-specific OXA β-lactamase is vague, but the fact of its existence itself raises concerns about its mobilization probability and further evolution in clinical *D. tsuruhatensis* strains. Nonetheless, the observed homologs of AMR genes were detected using genomics, and further experiments are needed to determine which specific genetic determinants are responsible for multidrug resistance in the *D. tsuruhatensis* strain MR-6/3H. Besides intrinsic genes, we also found acquired factors encoded by integrons in *the D. tsuruhatensis* TR1180 strain isolated from a human patient. TR1180 possessed *dfrA16* and *aadA3*, conferring resistance to trimethoprim and aminoglycosides, respectively. Both of them were the cassettes inside the complete integron sequence. These findings were consistent with the previously reported genomic investigation of TR1180 (Cheng et al., 2021). Integron class 3 possession was also reported in the *D. tsuruhatensis* A90 strain in 2007 (Xu et al., 2007). In 2021, Sun-Mi Cho et al. detected the integron class 1 in a clinical isolate of *D. tsuruhatensis*, which carried a *bla*_IMP-1_ gene with IMP-1 metallo-β-Lactamase production as well as some other integron-related AMR genes (Cho et al., 2021). It indicates the horizontal gene transfer (HGT) between *Delftia* spp. and other bacterial taxons. Therefore, *D. tsuruhatensis* can acquire mobile elements with AMR genes, which can cause the selection of resistant clones and rapid evolution of this bacterium, especially in the clinical environment. Moreover, one of the cassettes inside CALIN in the *D. tsuruhatensis* 161/1 strain matched with *Oreochromis niloticus* (fish) protein with an innate immune response function. Interestingly, there were also two cases of *D. tsuruhatensis* isolation from the abovementioned fish species, including isolation from an injured eye (Soto-Rodriguez et al., 2013; Preena et al., 2020). Such distant homology may indicate the ancient HGT evolutionary event between *Oreochromis niloticus* and *D. tsuruhatensis*. After transfer, this protein could be further used by bacteria to establish as a pathogen or symbiont in fish, depending on selection. To date, fewer cross-domain HGT (eukaryote-to-bacteria) events have been described (Dunning Hotopp, 2011).

Thus, the *D. tsuruhatensis* MR-6/3H strain isolated from raw milk and investigated in the present study was phylogenetically close to strains of clinical origin and possessed a common set of putative virulence factors with the latter ones. Raw milk can be the possible source of as well as a potential transmission route for the dissemination of MDR *D. tsuruhatensis*. However, the particular source of this isolate remains unclear. MR-6/3H also exhibited a multidrug-resistant phenotype, which was also consistent with performed resistome analysis. The carriage of a variety of genes encoding for efflux pumps indicates that the antimicrobial resistance mechanisms of *D. tsuruhatensis* are based mainly on the efflux of antibiotic molecules. However, considering the restrictions of the NGS approach, further experiments are needed to investigate which specific genes are responsible for antimicrobial resistance as well as for the pathogenicity of *D. tsuruhatensis*. In general, the *Delftia* genus requires a taxonomic revision, which was noted in the present as well as in some other studies. Additionally, considering the fact of ubiquity, high ecological plasticity, possession of some putative virulence factors, and intrinsic resistance determinants, *D. tsuruhatensis* has the great potency to succeed as an emergent nosocomial pathogen. Furthermore, it seems that *D. tsuruhatensis* readily acquires new genetic material via HGT, which can cause HGT-mediated rapid evolution, again, especially in the clinical environment. Further epidemiological investigation, particularly infection source detection and the development of clinical recommendations for antimicrobial therapy, are urgently required for the appropriate surveillance of *D. tsuruhatensis*.

## Materials and methods

### Bacterium isolation and cultural characteristics investigation

MR-6/3H was isolated from an unpasteurized bovine milk sample in the course of studies related to the screening for antibiotic-resistant bacteria in raw bovine milk. The milk sample was obtained from a retail seller in the Nizhny Novgorod district located in the European part of Russia in February 2022. Initially, 50 mL of milk sample was centrifuged at 8,000 rpm for 15 min to concentrate the bacterial biomass. The sediment was diluted in 2.5 mL of sterile phosphate buffer solution pH 7.2 (PBS, Himedia, Mumbai, India) and then aseptically added to 2.5 mL of tryptic soy broth (TSB, Himedia, Mumbai, India) supplemented with antibiotics. The following antibiotics were used in the final concentration: 50 μg/mL ampicillin, 25 μg/mL gentamicin, and 25 μg/mL kanamycin. Ampicillin was chosen as a beta-lactam representative with a comparatively wide antimicrobial spectrum (increased activity against gram-positive and some gram-negative species) (Wade and Benjamin, 2011). Gentamicin (II-aminoglycoside generation) and kanamycin (I-aminoglycoside generation) were used as aminoglycoside representatives to broader the spectrum due to the *in vitro* synergistic effect with ampicillin (Thieme et al., 2018). Additionally, 50 μg/mL fluconazole in the media was used to inhibit fungi growth. TSB was incubated for 48 h at 37°C in glass tubes without shaking. A loopful of broth was streaked onto tryptic soy agar (TSA, Himedia, Mumbai, India) media supplemented with the concentrations of antimicrobials noted above. The inoculated agar plate was incubated for 24 h at 37°C. The growth culture was further checked for purity through gram staining. Additionally, the OXITEST (Erba Lachema, Brno, Czechia) for oxidase activity detection and the 3% hydrogen peroxide test to identify catalase activity were carried out. To investigate hemolytic activity and cultural characteristics the 5% human O-blood agar was used (a blood sample was aseptically collected from a healthy adult volunteer with his documented consent; all procedures were performed in accordance with relevant guidelines and regulations). The stock culture was stored at-80°C in TSA (Himedia, Mumbai, India) with 15% glycerol.

### Isolate identification based on the 16S rRNA gene sequencing

Sequencing of a nearly full-length 16S rRNA gene was implemented for identification. Bacterial DNA was isolated via thermal lysis of cell suspension at 95°C for 20 min (a single colony from an agar plate was used). We performed a polymerase chain reaction with the following universal primers: 27F 5’-AGAGTTTGATCMTGGCTCAG-3′ and 1492R 5’-TACGGYTACCTTGTTACGACTT-3′ (Weisburg et al., 1991). PCR products of ~1,500 bp were detected in 1% agarose gel. Amplicons were extracted from the gel and purified using a DNA extraction kit (Dia-m, Moscow, Russia). Further DNA sequencing was performed using the ABI PRISM BigDye Terminator v. 3.1 reagent kit (PE Applied Biosystems, Foster City, CA) according to the manufacturer’s protocols, followed by an analysis of the reaction products on an automatic sequencer Applied Biosystems 3,730 DNA Analyzer. Obtained forward and reverse sequences were assembled into consensus using UGENE (v41.0) software (Okonechnikov et al., 2012). The EzTaxon server database was utilized to compare the resulting consensus to the sequences of other bacterial 16S rRNA genes (Yoon et al., 2017). To further clarify the strain identity, the average nucleotide identity (ANI) value, as well as DDH *in silico*, were calculated.

### Antimicrobial susceptibility via the Kirby-Bauer disk-diffusion method

The Kirby-Bauer disk-diffusion method (DDM) on Mueller-Hinton agar plates was performed according to the guidelines of EUCAST 2022 (the european committee on antimicrobial susceptibility testing). Breakpoint tables for interpretation of MICs and zone diameters, version 12.0, 2022,^1^ to assess the susceptibility profile of the MR-6/3H isolate were used. The following antibiotic disks were used: ampicillin (10 μg), amoxicillin-clavulanic acid (20–10 μg), aztreonam (30 μg), meropenem (10 μg), imipenem (10 μg), ceftobiprole (5 μg), cefotaxime (5 μg), cefepime (30 μg), ceftazidime (10 μg), piperacillin (30), piperacillin-tazobactam (100–10 μg), ticarcillin (75 μg), ticarcillin-clavulanic acid (75–10 μg), gentamicin (10 μg), amikacin (30 μg), tobramycin (10 μg), kanamycin (30 μg), neomycin (10 μg), ciprofloxacin (5 μg), levofloxacin (5 μg), chloramphenicol (30 μg), tetracycline (30 μg), and trimethoprim-sulfamethoxazole (1.25–23.75 μg). In total, the susceptibility to 23 antibacterials was tested. Mueller-Hinton media, as well as most antibiotic-containing disks, were obtained from HiMedia (Mumbai, India). Amoxicillin-clavulanate, gentamicin, polymyxin, and trimethoprim-sulfamethoxazole were obtained from NICF (Saint Petersburg, Russia). Aztreonam, piperacillin-tazobactam, ticarcillin, piperacillin, ticarcillin-clavulanate, tobramycin, and amikacin were obtained from Bioanalyse (Ankara, Turkey). Cefotaxime, cefepime, and ceftazidime were obtained from Erba Lachema (Brno, Czech Republic). As members of the *Delftia* genus are uncommon for clinical practice, EUCAST 2022 has no breakpoints for interpreting the results of DDM. In such cases, EUCAST recommends using the breakpoints of phylogenetically related bacteria. Therefore, we used *Burkholderia pseudomallei* and *Enterobacteriales* breakpoints to extend the number of tested antibiotics. *Escherichia coli* ATCC 25922 was used as a reference strain for quality control.

### Next-generation sequencing, assembly, and annotation

Genomic DNA was extracted from overnight MR-6/3H pure culture via the QIAamp DNA Kit (Qiagen, Hilden, Germany). The concentration of DNA was evaluated with the Qubit dsDNA BR Assay Kit on a Qubit 3.0 fluorometer (Fisher Scientific Inc., Waltham, MA, USA). Further Illumina Hiseq 1,500 (Illumina, CA, USA) paired-end (2×150) sequencing of *D. tsuruhatensis* MR-6/3H genome was performed by Geneanalytics LLC (Moscow, Russia). Raw read libraries were deposited at SRA (accession PRJNA903655).

Read library quality was evaluated using FastQC v0.11.9. Obtained reads were trimmed with Trimmomatic software (v0.4.8) using the automatic mode of adapter removal (Bolger et al., 2014). Unicycler v0.4.8. on the PATRIC resource center^2^ was utilized to assemble the genome (Brettin et al., 2015; Davis et al., 2020). Contigs less than 1,000 bp in length were removed from the resulting assembly. To assess the assembly quality, QUAST v5.0.2 and CheckM2 v1.0.2 tools were used (Gurevich et al., 2013; Chklovski et al., 2023). Genome annotation was performed at the Rapid Annotations using the Subsystems Technology (RAST) server (Aziz et al., 2008; Overbeek et al., 2014; Brettin et al., 2015) and the Prokaryote Genome Annotation Pipeline. The draft genome sequence has been deposited at GenBank (accession JAPMID000000000).

### Phylogenomic analysis

Phylogenomic analysis or phylogenomics in contrast to phylogenetic analysis (based on one or a small number of genes) implies the use the massive genomic data and demonstrates higher accuracy. Phylogenomics can be used to investigate evolutionary relationships between different taxons, accurate species identification, investigation of population structure, and pathogens epidemiology (Kapli et al., 2020).

Phylogenomic analysis of the MR-6/3H strain was performed for accurate species identification and population structure investigation. The list of strains used for phylogenomic analysis is shown in Supplementary Table S4. Strains were selected based on their origin, and members with missing origins were mostly excluded (except for type strains). *Comamonas testosteroni* TK102 was utilized as an outgroup. Genome-based species identification was carried out using the Average Nucleotide Identity Matrix calculator (ANI-matrix with ≥95% cutoff for species level) developed by Kostas lab, which estimates all-*vs*-all distances in a collection of genomes and Type (Strain) Genome Server (TYGS) (Rodriguez-R and Konstantinidis, 2016; Meier-Kolthoff and Göker, 2019). Phylogeny investigation was carried out with the Reference sequence Alignment based Phylogeny builder (REALPHY v1.13) with read length parameter 150 (RL = 150), which allows comparison between different species. *D. acidovorans* 2,167 (T), *D. lacustris* LMG 24775 (T), and *D. tsuruhatensis* NBRC 16741 (T) were used as reference genomes. The accuracy of REALPHY is comparable with the single-nucleotide polymorphism (SNP) approach. Moreover, REALPHY can consider more than one reference genome, which improves phylogeny reconstruction (Bertels et al., 2014). All cladograms were visualized and edited in iTOL v5 (Letunic and Bork, 2021).

### Comparative genomics

A comparative genomics approach was used to identify and compare putative antimicrobial resistance genes (AMR) and putative virulence factors (VFs). Comparative analysis was performed using the Galaxy Europe web server^3^ (Afgan et al., 2022). In all, 11 genomes of *Delftia* spp. strains with different origins, including *Delftia* MR-6/3H (Supplementary Table S1), were chosen to identify putative antimicrobial resistance genes and putative virulence factors. The strains mentioned above formed the common clade of *D. tsuruhatensis* and *D. lacustris* species and, therefore, were chosen for comparative analysis. AMR and VFs were predicted with BLASTp (v2.12.0) search against the Comprehensive Antibiotic Resistance Database (CARD v3.2.5) and virulence factor database set A (VFDB v2022.11.04) (Liu et al., 2019; Alcock et al., 2020). For both searches, the following cutoff parameters were used: e-value ≤1e-30, bitscore >100, query coverage ≥80%, and percentage of identity ≥60. The aforementioned parameters were chosen as recommended by W. Pearson for homology prediction but strengthened to avoid false-positive matching (Pearson, 2013).

Integrons were identified via IntegronFinder 2.0 with automatic parameters (Néron et al., 2022). Version 2.0 is upgraded and adapted to draft genome assemblies. IntegronFinder output consists of three types of integron: complete (include all *int* elements), In0 (integron-integrases lacking cassettes - include integrase only), and CALIN (clusters of *attC* sites lacking integron-integrases). Integron regions were visualized in Ugene v41.0 (Okonechnikov et al., 2012). A homology search of hypothetical proteins was performed via BLASTp (Madden et al., 1996) and HMMER web server (based on hidden Markov models (HMMs)) against the Reference Proteomes dataset (Potter et al., 2018).

### Prophage and CRISPR-Cas analysis

Bacteriophages or phages are known to be one of the major players contributing to the evolution of bacteria. Analysis of prophage genomes carriage allows us to investigate the evolution history of certain strains and analyze possible adaptive traits introduced by phage incorporation (Fortier and Sekulovic, 2013; Touchon et al., 2016). Prophage genomes and elements were detected using the PHASTER web tool with default parameters (Arndt et al., 2016). PHASTER uses a fasta assembly file as input and performs a BLAST search against a custom prophage/phage database. Detected phage elements were then clustered into prophage regions into three subgroups based on score: intact (score > 90), questionable (score from 70 to 90), and incomplete (score < 70). CRISPR-Cas elements were detected using the CRISPRCasFinder web tool with default parameters (Couvin et al., 2018).

## Data availability statement

The datasets presented in this study can be found in online repositories. The names of the repository/repositories and accession number(s) can be found in the article/Supplementary material.

## Ethics statement

The studies involving humans were approved by Ethics committee of Federal Research Center for Virology and Microbiology. The studies were conducted in accordance with the local legislation and institutional requirements. The participants provided their written informed consent to participate in this study.

## Author contributions

PA: Conceptualization, Formal analysis, Methodology, Project administration, Supervision, Visualization, Writing – original draft, Writing – review & editing. DK: Investigation, Validation, Writing – review & editing. AM: Investigation, Validation, Writing – review & editing.

## Footnotes

^1^ https://www.eucast.org/

^2^ https://patricbrc.org/

^3^ https://usegalaxy.eu
