# Supplementary figures and images for: Genomic analysis of multidrug-resistant Delftia tsuruhatensis isolated from raw bovine milk
Source: Front Microbiol. 2024 Jan 4;14:1321122. doi: 10.3389/fmicb.2023.1321122 (PMC10794605; doi:10.3389/fmicb.2023.1321122)

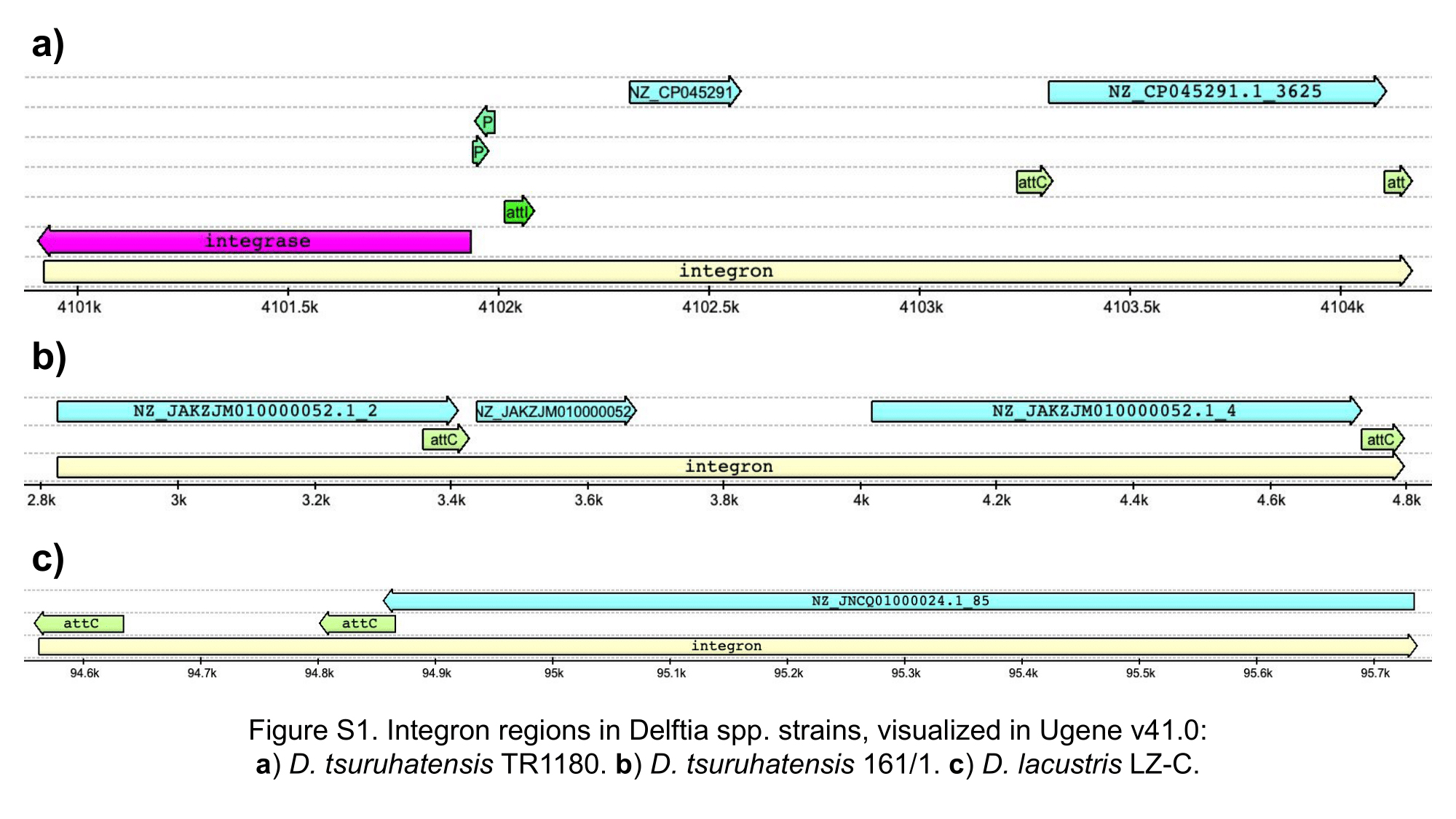

Supplement: Supplementary file 5 [file Image_1.JPEG]
